# Supplementary material for: Mechanically enhanced biogenesis of gut spheroids with instability-driven morphomechanics
Source: Nat Commun. 2023 Sep 27;14:6016. doi: 10.1038/s41467-023-41760-2 (PMC10533890; doi:10.1038/s41467-023-41760-2)
Supplement: Supplementary file 1 — Supplementary Information (Single PDF) [file 41467_2023_41760_MOESM1_ESM.pdf]

Supplementary Information

Mechanically enhanced biogenesis of gut spheroids with instability-driven morphomechanics

Feng Lin[1,3,†], Xia Li[1,†], Shiyu Sun[1,4,†], Zhongyi Li[1,†], Chenglin Lv[1], Jianbo Bai[1], Lin Song[2], Yizhao Han[1], Bo Li[1,*], Jianping Fu[4,5,6], and Yue Shao[1,2,*]

[1] Institute of Biomechanics and Medical Engineering, Department of Engineering Mechanics, School of Aerospace Engineering, Tsinghua University, Beijing 100084, China; [2] State Key Laboratory of Primate Biomedical Research, Institute of Primate Translational Medicine, Kunming University of Science and Technology, Kunming, Yunnan 650500, China; [3] Wenzhou Institute, University of Chinese Academy of Sciences, Wenzhou, Zhejiang 325000, China; [4] Department of Mechanical Engineering, University of Michigan, Ann Arbor, MI 48109, USA; [5] Department of Biomedical Engineering, University of Michigan, Ann Arbor, MI 48109, USA; [6] Department of Cell & Developmental Biology, University of Michigan Medical School, Ann Arbor, MI 48109, USA

[†]These authors contributed equally to this work;

* Correspondence should be addressed to Y. Shao (yshao@tsinghua.edu.cn) and B. Li (libome@tsinghua.edu.cn).

This document contains:

Supplementary Figures and Captions

Supplementary Tables

Supplementary Figures and Captions

Supplementary Figure 1

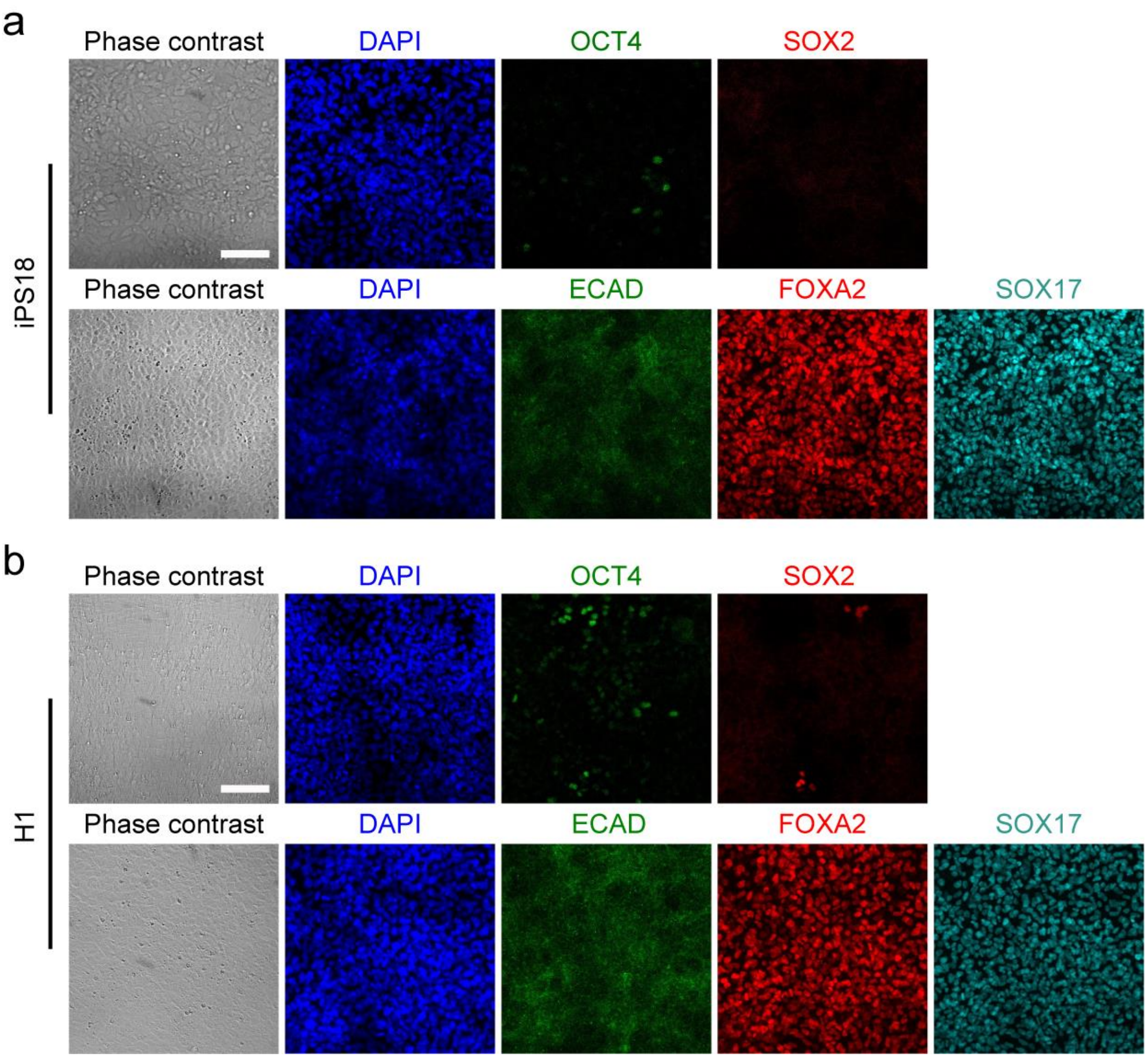

Supplementary Figure 1. Definitive endoderm cells differentiated from human pluripotent stem cells. Representative phase contrast images of DE cells on day 4, and confocal micrographs showing the staining of OCT4, SOX2, E-cadherin (ECAD), FOXA2, and SOX17 in DE cells derived from iPS18 **(a)** and H1 **(b)** cells. DAPI stains cell nuclei. Scale bar: 100 µm. *n* = 3 independent experiments.

Supplementary Figure 2

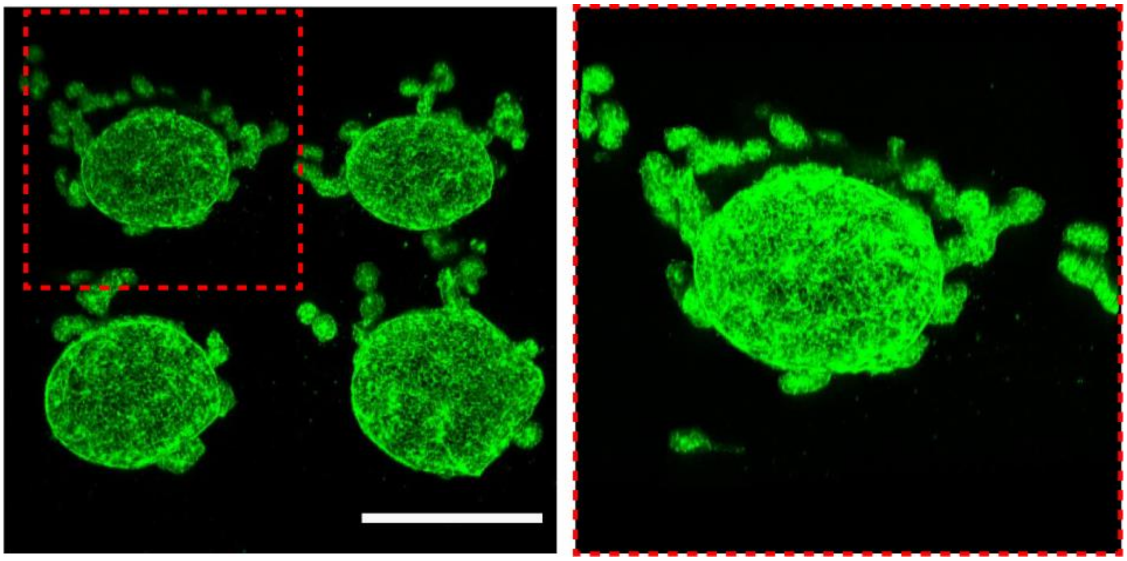

Supplementary Figure 2. Tissue columns emerged from originally flat colony in micropatterned gut spheroid generator (μGSG). Representative 3D-reconstructed confocal micrographs showing the staining of F-actin in day 7 cell culture in μGSG. Dashed red rectangle marks the single-colony shown in zoom-in image. Notable tissue columns were formed across the colony. Further “pearling” of the tissue column, visible as the tissue columns transformed into interconnected “tissue buds”, was also observed. Micropattern diameter: 400 μm. Scale bar: 400 μm. *n* = 3 independent experiments.

Supplementary Figure 3

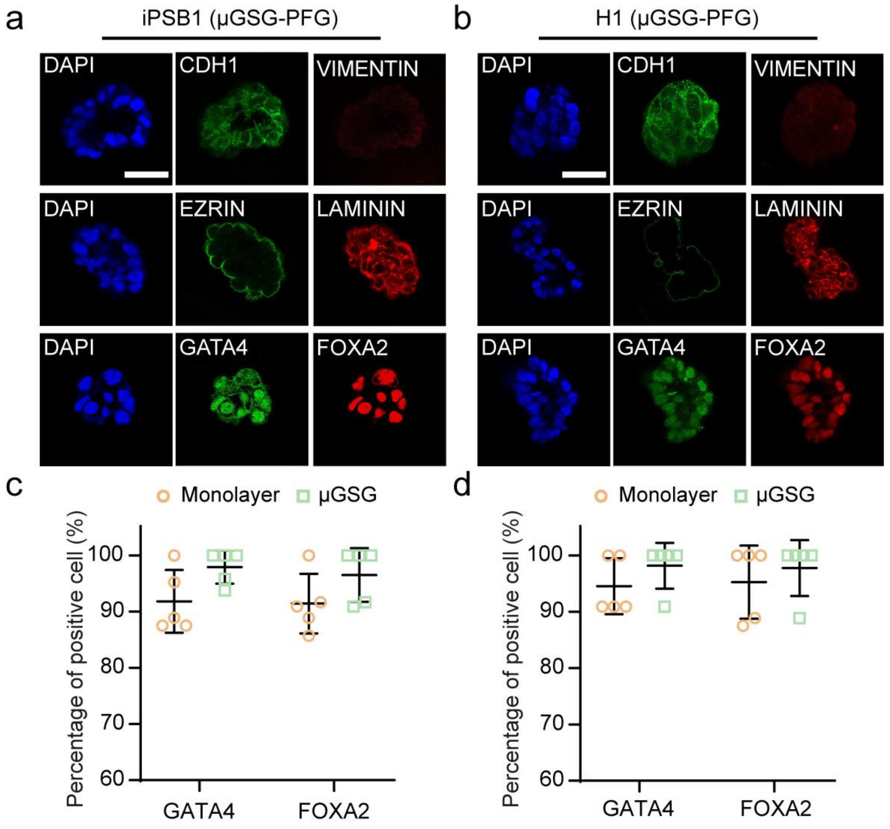

Supplementary Figure 3. Characterization of PFG (posterior foregut) spheroids derived from μGSG (micropatterned gut spheroid generator) using iPSB1 and H1 cell lines. (a&b) Representative confocal micrographs showing the staining of EZRIN, LAMININ, CDH1, VIMENTIN, GATA4, and FOXA2 in μGSG-induced PFG spheroids using iPSB1 **(a)** and H1 **(b)** cell lines. Similar results were seen in n = 5 independent experiments. **(c&d)** Scatter plots showing percentages of cells positive for GATA4 and FOXA2 in PFG spheroids derived from indicated conditions using iPSB1 **(c)** and H1 **(d)** cell lines. Scale bar: 50 μm. n = 5 independent experiments. All data were plotted as mean ± s.d. Source data are provided as a Source Data file.

Supplementary Figure 4

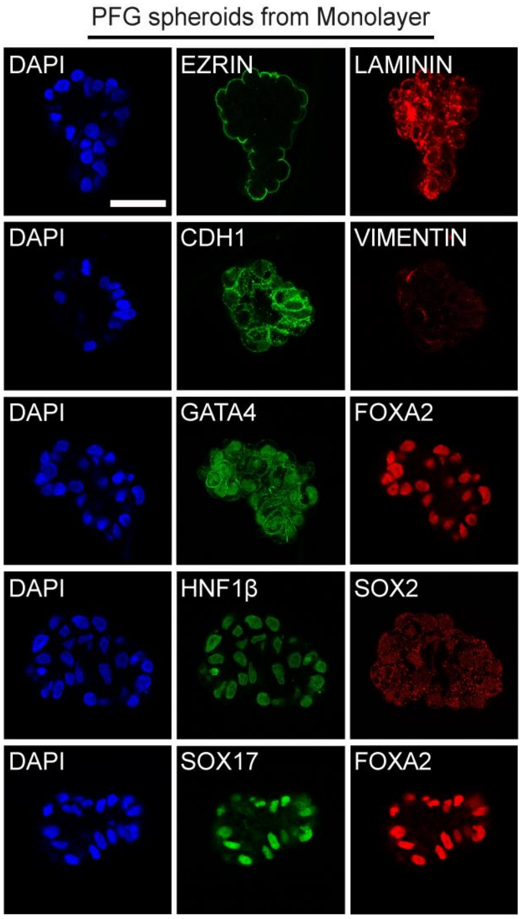

Supplementary Figure 4. Characterization of PFG (posterior foregut) spheroids generated from monolayer-based induction. Representative confocal micrographs showing the staining of DAPI, EZRIN, LAMININ, CDH1, VIMENTIN, GATA4, FOXA2, HNF1β, SOX2, and SOX17 in PFG spheroids generated by monolayer-based induction using iPS18 cells. Scale bar: 50 μm. Similar results were seen in *n* = 5 independent experiments.

Supplementary Figure 5

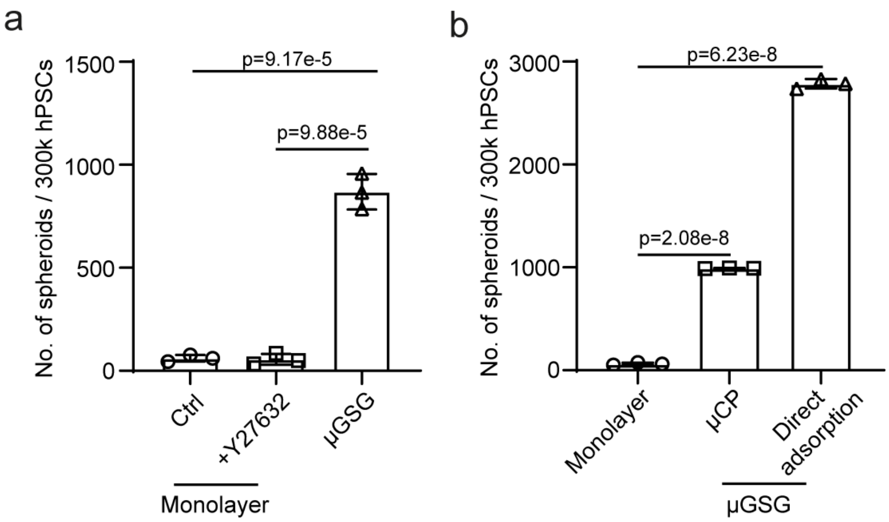

Supplementary Figure 5. μGSG (micropatterned gut spheroid generator) enhances the biogenesis of gut spheroids independent of transient ROCK inhibition and substrate preparation methods. (a) Bar plot showing the number of PFG (posterior foregut) spheroids generated on day 7 using monolayer-induction with transient treatments of DMSO (Ctrl) or 5 μm Y27632 from day 4 to 5, in comparison to the number of spheroids generated using μGSG. *n* = 3 independent experiments. **(b)** Bar plot showing the number of PFG spheroids generated on day 7 using μGSG with substrates prepared by micro-contact printing (μCP) and direct adsorption, in comparison to the number of spheroids generated using monolayer-induction. *n* = 3 independent experiments. All data were plotted as mean ± s.d. *P*-values were calculated using unpaired, two-sided Student's t-test. Source data are provided as a Source Data file.

Supplementary Figure 6

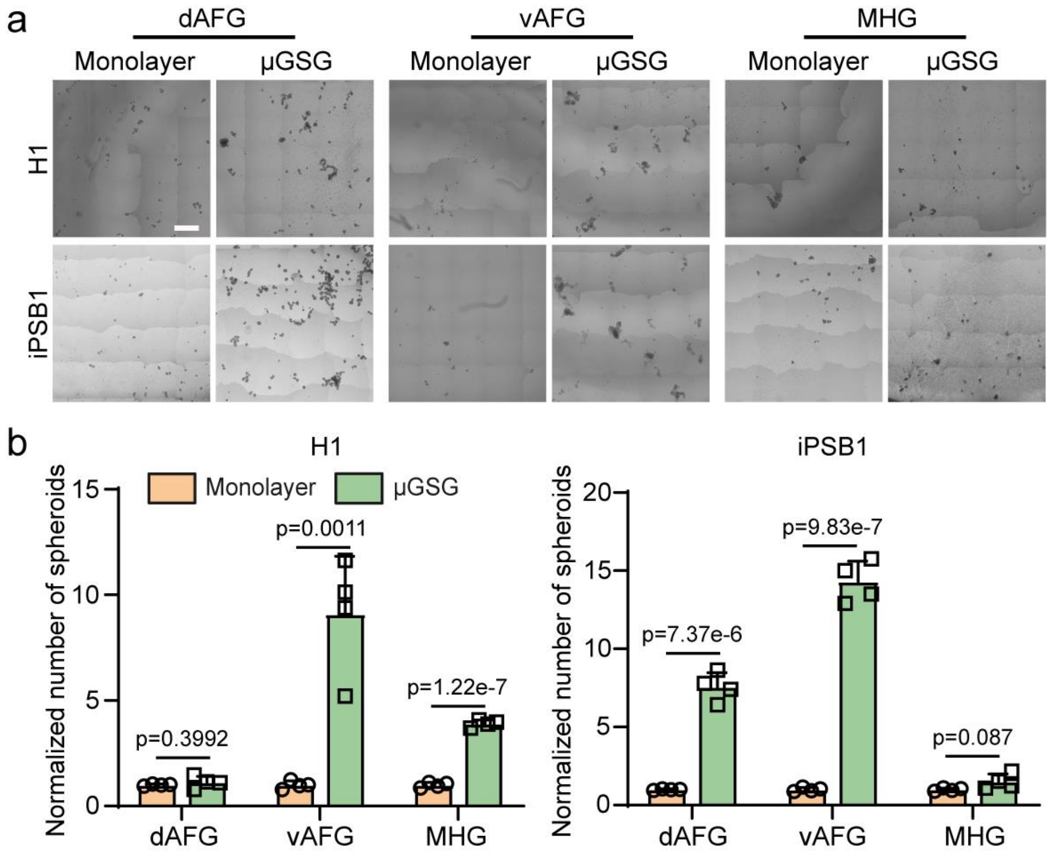

Supplementary Figure 6. Efficient generation of different types of region-specific gut spheroids from μGSG (micropatterned gut spheroid generator) using H1 and iPSB1 cell lines. (a) Representative phase contrast images showing dAFG (dorsal anterior foregut), vAFG (ventral anterior foregut), and MHG (mid-hind gut) spheroids collected from monolayer-induction and μGSG using H1 (upper) and iPSB1 (lower) cell lines. Scale bar: 500 μm. Similar results were seen in *n* = 4 independent experiments. **(b)** Bar plots showing normalized number of dAFG, vAFG and MHG spheroids collected from monolayer-induction and μGSG using H1 (left) and iPSB1 (right) cell lines. *n* = 4 independent experiments. All data were plotted as mean ± s.d. *P*-values were calculated using unpaired, two-sided Student’s t-test. Source data are provided as a Source Data file.

Supplementary Figure 7

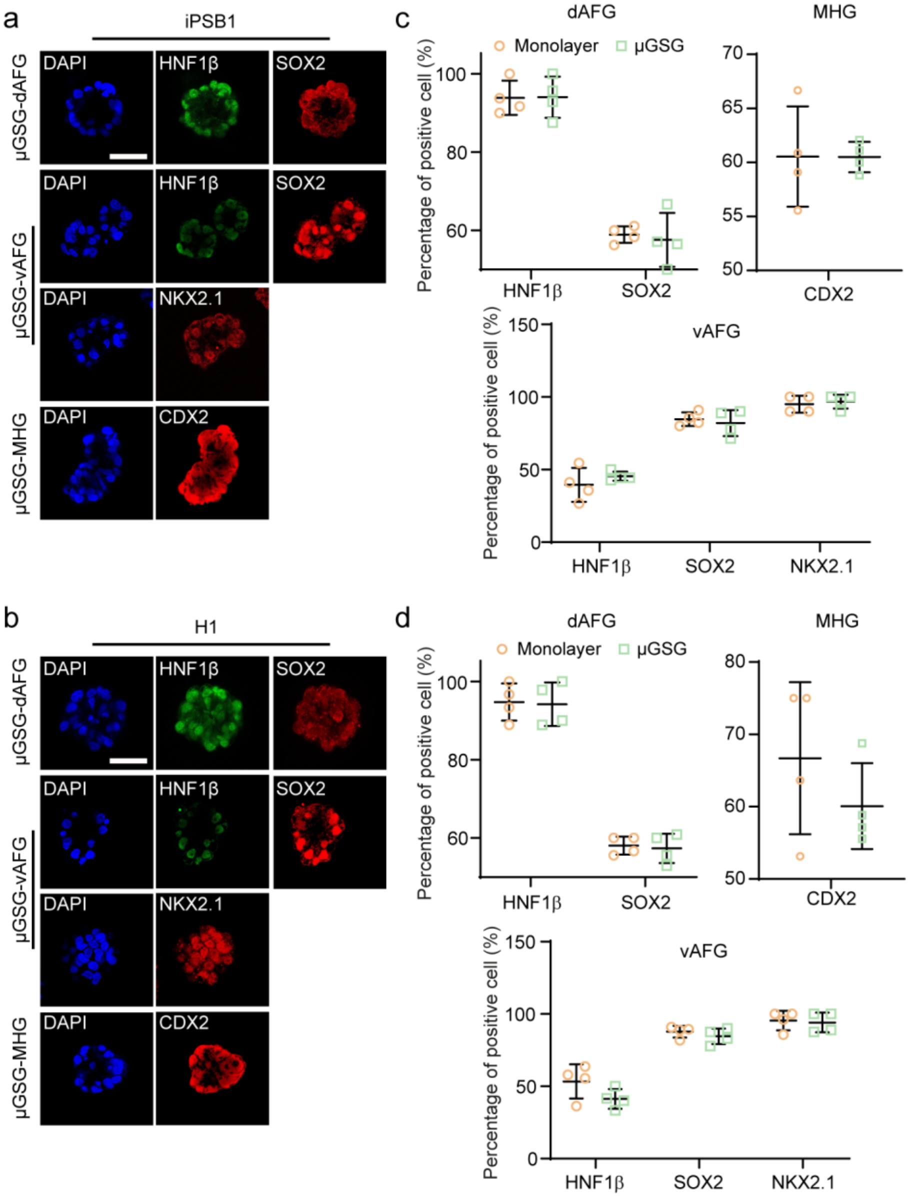

Supplementary Figure 7. Characterization of different types of region-specific gut spheroids derived from µGSG (micropatterned gut spheroid generator) using iPSB1 and H1 cell lines. (a&b) Representative confocal micrographs showing immunostaining of HNF1β and SOX2 in µGSG-derived dAFG (dorsal anterior foregut) spheroids, HNF1β, SOX2, and NKX2.1 in µGSG-derived vAFG (ventral anterior foregut) spheroids, and CDX2 in µGSG-derived MHG (mid-hind gut) spheroids, using iPSB1 **(a)** and H1 **(b)** cell lines. DAPI counterstains cell nuclei. Similar results were seen in n = 4 independent

experiments. **(c&d)** Scatter plots showing percentages of cells positive for HNF1β and SOX2 in dAFG spheroids, HNF1β, SOX2, and NKX2.1 in vAFG spheroids, and CDX2 in MHG spheroids generated from indicated conditions using iPSB1 **(c)** and H1 **(d)** cell lines. Scale bar: 50 μm. All data were plotted as mean ± s.d. n = 4 independent experiments. Source data are provided as a Source Data file.

Supplementary Figure 8

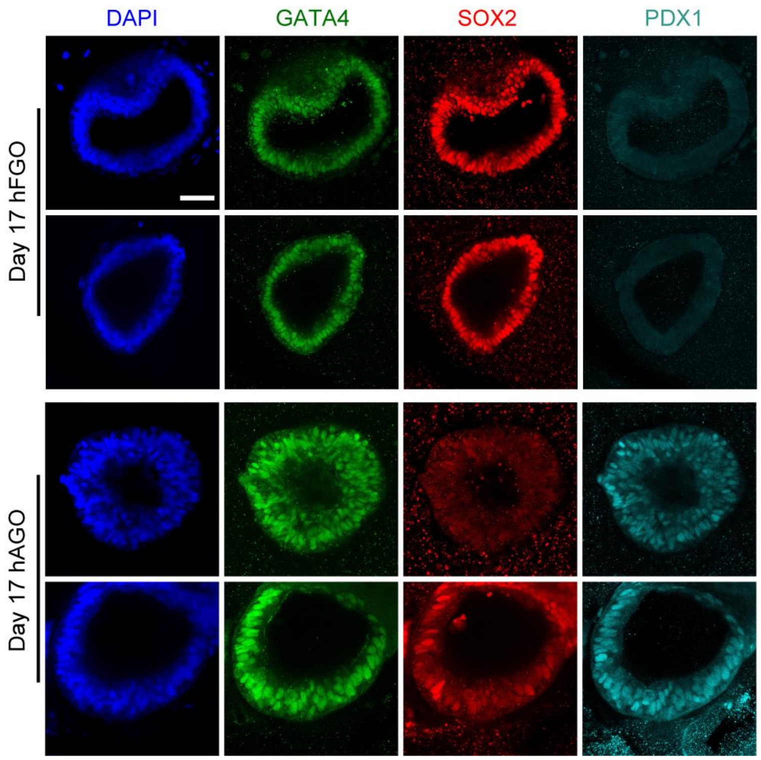

Supplementary Figure 8. Gastric organoids generated from monolayer-induced PFG (posterior foregut) spheroids. Confocal micrographs showing immunostaining of GATA4, SOX2, and PDX1 in day 17 hFGO (human fundic gastric organoid, upper) and hAGO (human antral gastric organoid, lower) generated from monolayer-induced PFG spheroids. DAPI stains the nuclei. Scale bars, 100 µm. $n = 3$ independent experiments.

Supplementary Figure 9

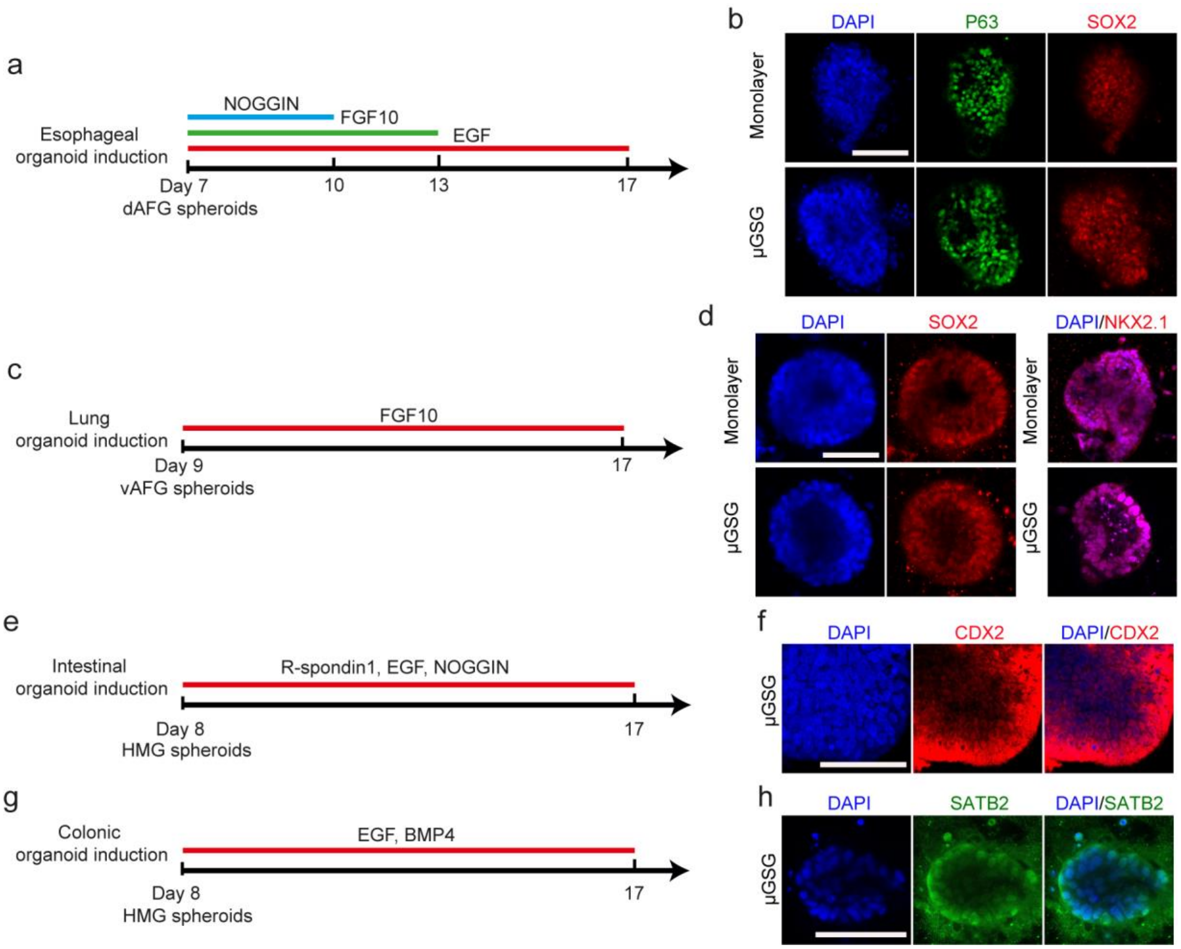

Supplementary Figure 9. Different type of region-specific gut spheroids generated from μGSG (micropatterned gut spheroid generator) possess region-specific developmental potential. (a) Protocol for esophageal organoid induction. **(b)** Confocal micrographs showing the staining of P63 and SOX2 in day 17 esophageal organoids generated using dAFG (dorsal anterior foregut) spheroids induced from indicated conditions. DAPI stains cell nuclei. *n* = 3 independent experiments. **(c)** Protocol for lung organoid differentiation. **(d)** Confocal micrographs showing the staining of SOX2 and NKX2.1 in day 17 lung organoids generated using vAFG (ventral anterior foregut) spheroids induced from indicated conditions. DAPI stains cell nuclei. *n* = 3 independent experiments. **(e)** Protocol for intestinal organoid differentiation. **(f)** Confocal micrographs showing the staining of cell nuclei (DAPI) and CDX2

in day 17 intestinal organoids generated using μGSG-derived MHG (mid-hind gut) spheroids. *n* = 3 independent experiments. **(g)** Protocol for colonic organoids differentiation. **(h)** Confocal micrographs showing the staining of cell nuclei (DAPI) and SATB2 in day 17 colonic organoids generated using μGSG-derived MHG spheroids. Scale bar: 100 μm. *n* = 3 independent experiments.

Supplementary Figure 10

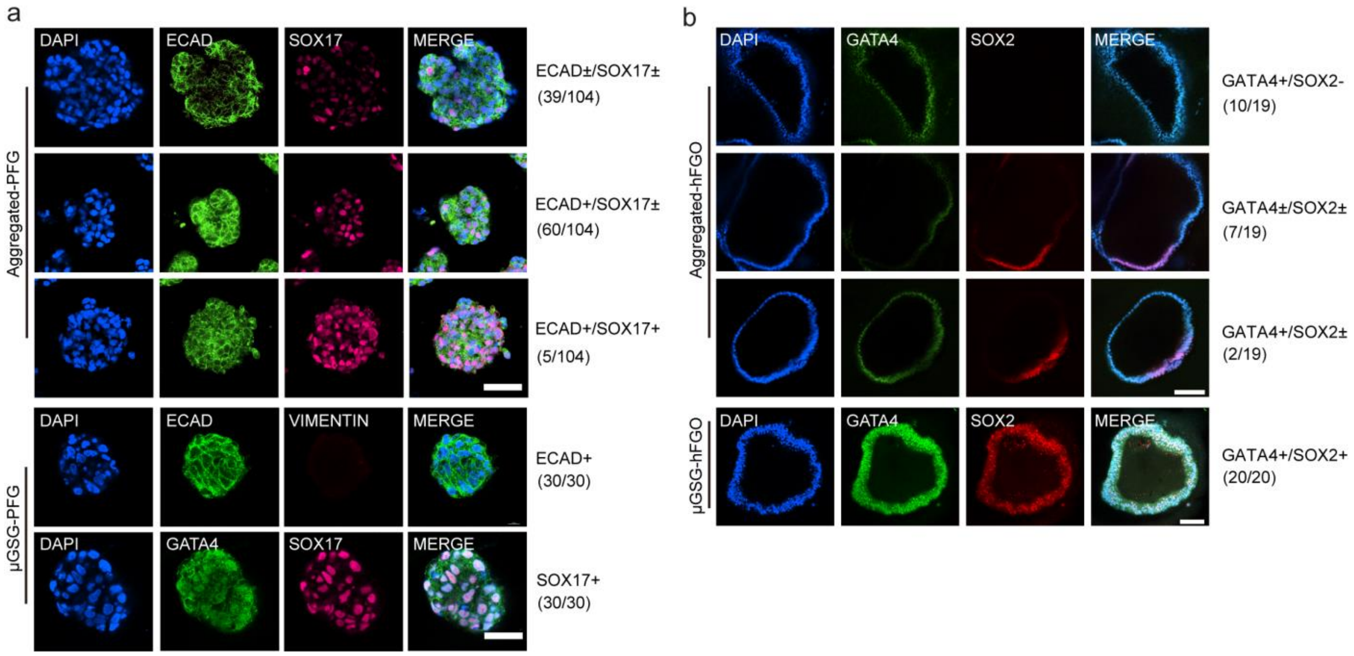

Supplementary Figure 10. Limited biological fidelity of PFG (posterior foregut) and hFGO (human fundic gastric organoid) generated by forced cell aggregation. (a) Confocal micrographs showing immunostaining of E-cadherin (ECAD) and SOX17 in PFG spheroids made by forced cell aggregation (upper), as well as ECAD, VIMENTIN, GATA4, and SOX17 in micropatterned gut spheroid generator (μGSG)-PFG spheroids (lower). DAPI stains the nuclei. *n* =3 independent experiments. The denominator reflects the total number of PFG spheroids quantitated, while the numerator is the number of indicated PFG phenotype among the quantitated PFG spheroids. Scale bar: 50 μm. **(b)** Confocal micrographs showing immunostaining of GATA4 and SOX2 in day 17 hFGO generated from PFG spheroids made by forced cell aggregation (upper), as well as in day 17 μGSG-PFG (lower). DAPI stains the nuclei. *n* = 3 independent experiments. The denominator reflects the total number of hFGO quantitated, while the numerator is the number of indicated hFGO phenotype among the quantitated hFGO. Scale bars, 100 μm.

Supplementary Figure 11

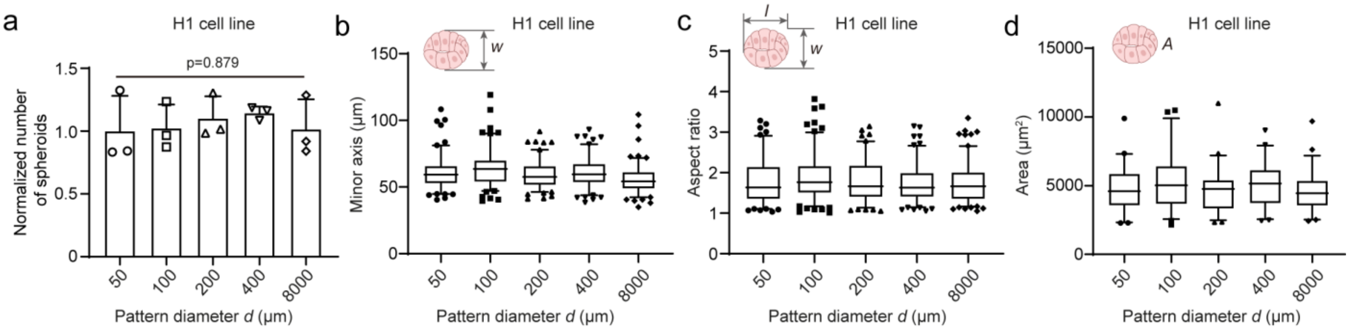

Supplementary Figure 11. Spheroid morphogenesis from μGSG (micropatterned gut spheroid generator) using H1 cell line is independent of micropattern sizes. (a) Bar blot showing normalized number of PFG (posterior foregut) spheroids collected from μGSG featuring different micropattern diameters using H1 cell line. Data were plotted as mean ± s.d. *n* = 3 independent experiments. *P*-values were calculated using one-way analysis of variance (ANOVA) and unpaired, two-sided Student's t-test. **(b-d)** Box charts showing minor axis length, w **(b)**, aspect ratio, l/w **(c)**, and projected area, A **(d)** of PFG spheroids derived from μGSG using H1 cell line (box: 25% - 75%, bar-in-box: median, and whiskers: 5% and 95%). *n* = 3 independent experiments. $n_{spheroids}$ = 50 - 152 for each independent experiment. Source data are provided as a Source Data file.

Supplementary Figure 12

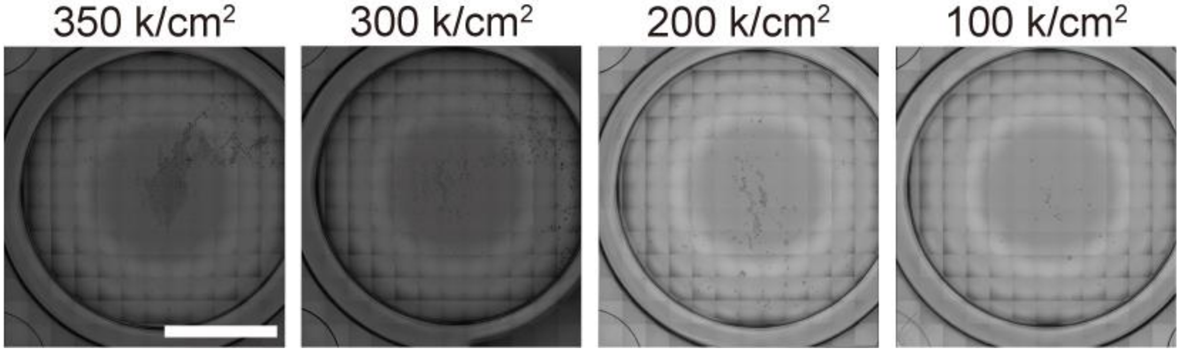

Supplementary Figure 12. Generation of PFG (posterior foregut) spheroids from μGSG (micropatterned gut spheroid generator) using different plating density of DE (definitive endoderm) cells. Representative phase contrast images showing all PFG spheroids collected from μGSG using indicated plating density of DE cells. Similar results were observed in *n* = 3 independent experiments. Scale bar: 8000 μm.

Supplementary Figure 13

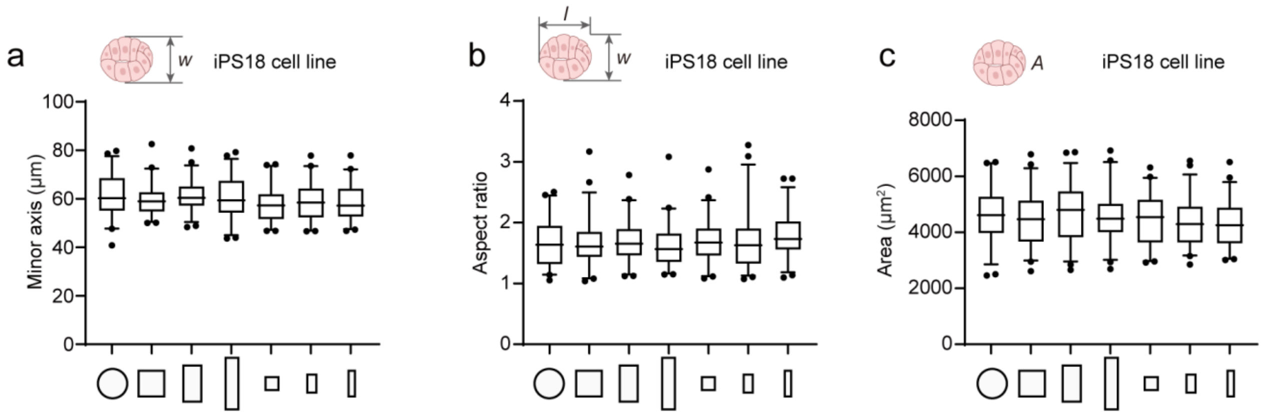

Supplementary Figure 13. Conserved morphological features of gut spheroids generated by μGSG (micropatterned gut spheroid generator) of different shapes and sizes. Box charts showing the minor axis length, w **(a)**, aspect ratio, l/w **(b)**, and projected area, A **(c)** of posterior foregut spheroids generated from indicated conditions (box: 25% - 75%, bar-in-box: median, and whiskers: 5% and 95%). Circular μGSG (d = 400 μm), large rectangular micropatterns (Aspect ratio = 1:1, 1:2, and 1:4; Area = 1.26×10^5 μm^2), small rectangular micropatterns (Aspect ratio = 1:1, 1:2, and 1:4; Area = 3.14×10^4 μm^2). n = 3 independent experiments. $n_{spheroids}$ = 46 - 54 for each independent experiment. Source data are provided as a Source Data file.

Supplementary Figure 14

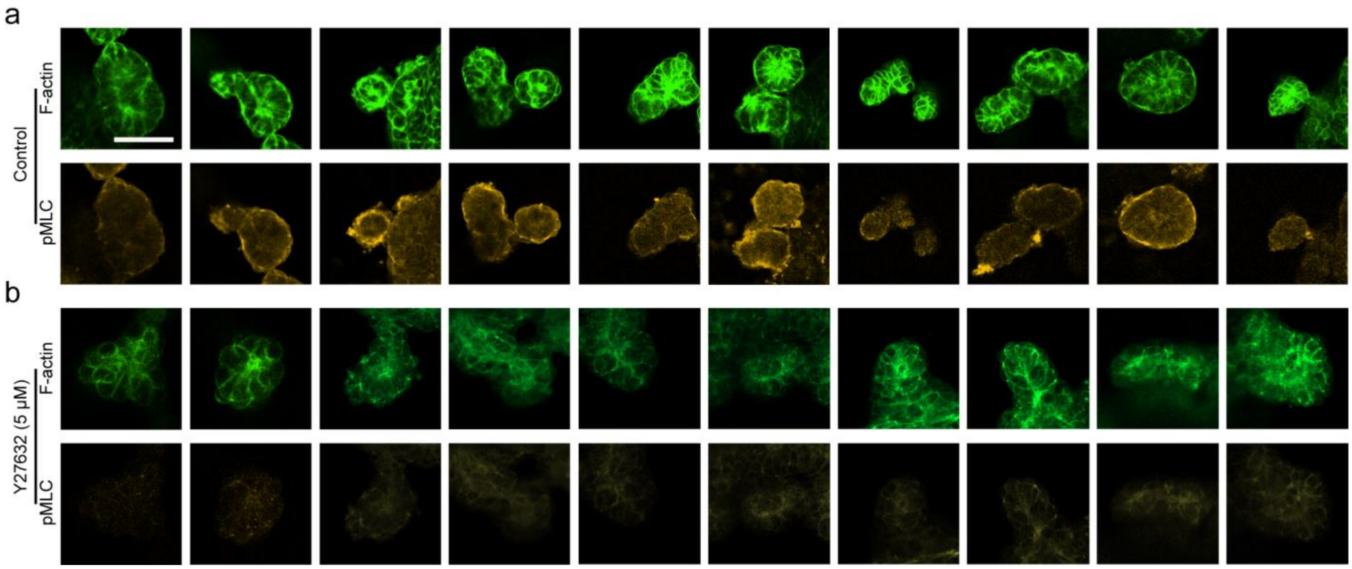

Supplementary Figure 14. Mechanosensitive accumulation of actomyosin machinery at outer tissue surfaces in pre-fission tissue columns / buds. Confocal micrographs showing the staining of F-actin and pMLC (phosphorylated myosin light chain) in pre-fission tissue columns / buds formed in micropatterned gut spheroid generator on day 7 under control **(a)** and 5 μM Y27632 treatment **(b)** conditions. Similar results were observed in n = 3 independent experiments. Scale bar: 50 μm.

Supplementary Figure 15

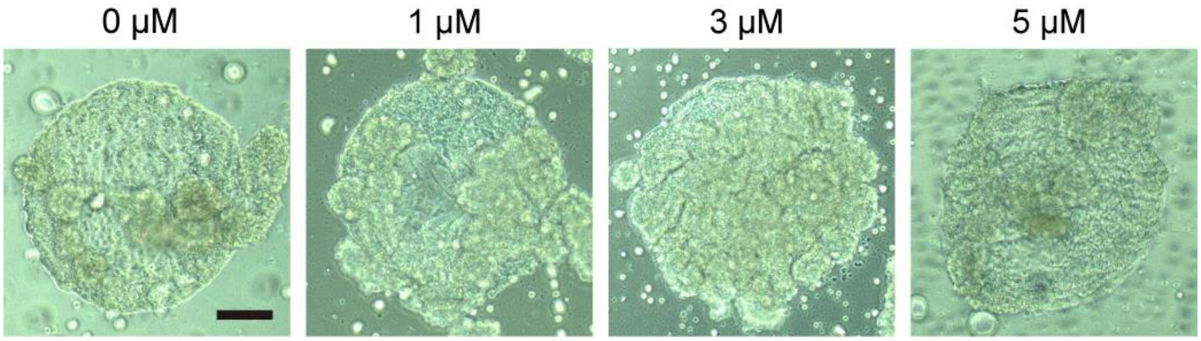

Supplementary Figure 15. Remnant tissue "pearling" under Y27632 treatment. Representative phase contrast images showing posterior foregut tissues that remain attached in micropatterned gut spheroid generator on day 7 under Y27632 treatment with different concentrations. Scale bar: 100 µm. Similar results were observed in n = 3 independent experiments.

Supplementary Table 1. List of primary antibodies used in immunocytochemistry (ICC).

Protein	Species	Application	Catalog No.	Vendor
Primary antibodies				
EZRIN	Mouse	1:200	E8897	Sigma-Aldrich
E-CADHERIN	Mouse	1:200	610181	BD Biosciences
pMLC	Rabbit	1:100	3671S	Cell Signaling Technology
OCT4	Mouse	1:200	SC-5279	Santa-Cruz Biotechnology
SOX2	Rabbit	1:800	Ab97959	Abcam
SOX17	Goat	1:200	AF1924	R&D
GATA4	Mouse	1:500	SC-25310	Santa-Cruz Biotechnology
HNF1β	Mouse	1:500	612504	BD Transduction Laboratories
LAMININ	Rabbit	1:50	Ab11575	Abcam
VIMENTIN	Rabbit	1:100	5741S	Cell Signaling Technology
FOXA2	Rabbit	1:400	8186S	Cell Signaling Technology
NKX2.1	Rabbit	1:100	Ab76013	Abcam
SATB2	Mouse	100 μg/mL	Ab51502	Abcam
CDX2	Rabbit	1:200	Ab76541	Abcam
PDX1	Goat	1:2000	Ab47383	Abcam
P63	Mouse	1:100	Ab735	Abcam
CLDN18	Rabbit	1:100	HPA018446	HPA
Secondary antibodies				
Anti-Mouse 488	Donkey	1:500	A21202	Thermo Fisher Scientific
Anti-Rabbit 568	Donkey	1:500	A10042	Thermo Fisher Scientific
Anti-Goat 647	Donkey	1:500	A32849	Thermo Fisher Scientific
