# Supplementary material for: Mechanically enhanced biogenesis of gut spheroids with instability-driven morphomechanics
Source: Nat Commun. 2023 Sep 27;14:6016. doi: 10.1038/s41467-023-41760-2 (PMC10533890; doi:10.1038/s41467-023-41760-2)
Supplement: Supplementary file 4 — Description of Additional Supplementary files [file 41467_2023_41760_MOESM4_ESM.pdf]

Description of Additional Supplementary files

File name: Supplementary Movie 1.
Description: 3D-reconstructed timelapse movie showing the morphogenesis of a PFG spheroid in μGSG from day 6-7.

File name: Supplementary Movie 2.
Description: Simulation results showing the dynamic process of spheroid morphogenesis. Dimensionless timestamp is also shown in this movie.
